# Under cover of the night: context-dependency of anthropogenic disturbance on stress levels of wild roe deer *Capreolus capreolus*

**DOI:** 10.1093/conphys/coaa086

**Published:** 2020-09-22

**Authors:** Jeffrey Carbillet, Benjamin Rey, Rupert Palme, Nicolas Morellet, Nadège Bonnot, Yannick Chaval, Bruno Cargnelutti, A J M Hewison, Emmanuelle Gilot-Fromont, Hélène Verheyden

**Affiliations:** 1 INRAE, CEFS, Université de Toulouse, F-31326, Castanet Tolosan, France; 2 VetAgro Sup Campus vétérinaire de Lyon, Université de Lyon, F-69280, Marcy-l’Etoile, France; 3 Laboratoire de Biométrie et Biologie Evolutive UMR 5558, CNRS, Université Lyon 1, Université de Lyon, F-69622 Villeurbanne, France; 4 Unit of Physiology, Pathophysiology, and Experimental Endocrinology, Department of Biomedical Sciences, University of Veterinary Medicine, Vienna, 1210, Austria; 5 EFNO, INRAE, F-45290, Nogent-sur-Vernisson, France

**Keywords:** Faecal glucocorticoid metabolites, human disturbance, space use, stress hormones

## Abstract

Wildlife populations are increasingly exposed to human-induced modifications of their habitats. To cope with anthropogenic stressors, animals can adjust their behaviour—for example, by shifting their activity to more sheltered habitats, or becoming more nocturnal. However, whether use of spatial and temporal adjustments in behaviour may regulate the endocrine response is poorly documented. Here, we analyzed faecal cortisol metabolites (FCMs) of wild roe deer (*Capreolus capreolus*) living in a human-dominated agro-ecosystem. Using Global Positioning System monitoring of 116 individuals, we assessed their spatial behaviour and tested whether proximity to anthropogenic structures (linear distance to built-up areas) and the use of refuge habitats (woodland and hedges) influenced FCM levels. In accordance with our predictions, individuals ranging closer to anthropogenic structures during daytime had higher FCM levels, but this relationship was buffered as use of refuge habitat increased. In addition, this link between proximity to anthropogenic structures and FCM levels disappeared when we analyzed spatial behaviour at night. Finally, FCM levels were higher when the ambient temperature was lower, and during years of low resource availability. Our results demonstrate that the stress levels of large mammals may be strongly influenced by their proximity to anthropogenic activities, but that these effects may be buffered by behavioural adjustments in terms of space use and circadian rhythm. Whereas most studies have focused on the influence of environmental heterogeneity, our analysis highlights the need to also consider the fine-scale spatial response of individuals when studying the hormonal response of wild animals to human disturbance. We emphasize the potential to mitigate this hormonal stress response, and its potential negative consequences on population dynamics, through the preservation or restoration of patches of refuge habitat in close proximity to human infrastructure.

## Teaser

We tested whether proximity to sources of anthropogenic disturbance influenced baseline stress levels of a wild ungulate in relation to their space use behaviour. We found that faecal cortisol metabolites increased as distance to human infrastructure decreased, but this relationship was buffered by the use of refuge habitats during daytime.

## 1. Introduction

The intensification of anthropogenic activities over recent decades, particularly since the 1950s, lead Crutzen and Stoermer (2000) and Steffen *et al*. (2007) to call the modern era the ‘Anthropocene’. Changes in land use, particularly within agricultural areas, has resulted in landscape modifications and habitat fragmentation over more than 50% of the earth’s land surface (Hooke *et al*., 2012). Wild animals are, therefore, increasingly exposed to human-induced modifications of their habitats that can have consequences for ecosystem processes through the modification and restriction of their movements and space use (Tucker *et al*., 2018). For example, large mammals, and particularly large herbivores, usually occupy a large home range and are widely hunted, so that they are particularly vulnerable to the consequences of human activities, including landscape fragmentation. These habitat modifications may act as chronic stressors since large mammals usually do not easily adapt to erratic and unpredictable environmental variations, such as those associated with human activities (Romero and Wingfield, 2015).

Glucocorticoids are metabolic hormones that ensure the regulation of an individual’s energy balance though acquisition, storage and mobilization (Landys *et al*., 2006; MacDougall-Shackleton *et al*., 2019). In response to unpredictable stimuli, the activation of the hypothalamic-pituitary-adrenal (HPA) axis usually results in increased secretion and release of glucocorticoids (cortisol and corticosterone) into the blood stream by the adrenal gland cortex (Sapolsky *et al*., 2000; Reeder and Kramer, 2005). The increase in glucocorticoid concentration supports the action of catecholamines by inhibiting energy storage to mobilize lipids and glucose, promote cardiovascular function, increase locomotor activity (Hau *et al*., 2016; Palme 2019) and induce a redistribution of immune cells (Dhabhar and McEwen, 1997). When stressors persist or recur, glucocorticoid regulation becomes less effective, and glucocorticoid secretion remains elevated for longer. This physiological state, referred to as chronic stress, is often associated with negative consequences (McEwen and Wingfield, 2010), including a decreased immune response and increased susceptibility to disease (Dhabhar 2014), reduced growth (Busch *et al*., 2008) and a decrease in reproductive performance (Sapolsky *et al*., 2000). These adverse effects can impact individual fitness (Romero *et al*., 2009) and, ultimately, population dynamics (Wingfield and Sapolsky, 2003).

Several recent studies suggest that animals living in disturbed habitat had higher glucocorticoid levels than those living in undisturbed areas (Dantzer *et al*., 2014; Rehnus *et al*., 2014; Jachowski *et al*., 2015; Formenti *et al*., 2018). However, individuals may become habituated to human activity, leading to blunting of the stress response (e.g. urban environments (Romero *et al*., 2009; Fokidis *et al*., 2009). In addition to elevated glucocorticoid levels, fear associated with human disturbance may also drive animals to modify their space use in order to reduce exposure to such stressors [Jachowski *et al*., 2012 on African elephants (*Loxodonta africana*); Martin *et al*., 2010 on the brown bear (*Ursus arctos*); Thiel *et al*., 2008 on capercaillie (*Tetrao urogallus*)]. In the landscape of fear framework (Laundré *et al*., 2001), animals are, therefore, predicted to adjust their spatial behaviour to spatio-temporal variations in the perceived risk of predation or disturbance. For instance, Martin *et al*. (2018) showed that roe deer (*Capreolus capreolus*) confined their movements to safe habitats during daytime and during the hunting season, when human activity is high. To minimize exposure to anthropogenic stressors, wild animals also tend to shift their activity patterns to become more nocturnal (Gaynor *et al*., 2018). However, whether use of these spatial and temporal adjustments in behaviour may regulate the endocrine response remains poorly documented. Yet, these behavioural tactics may attenuate the physiological stress response and, hence, reduce the risk of chronic adverse effects of high glucocorticoid levels.

Here, we analyzed variation in faecal cortisol metabolites (FCMs) of roe deer from a wild population living in a heterogeneous landscape composed of agricultural fields providing abundant forage interspersed with woodlands and hedgerows that can be considered as refuge habitat, in addition to providing food resources. Arguably, measuring FCMs is a useful way of assessing the baseline or cumulative levels of stress to which an individual has been exposed. FCMs represent an integrative measure that dampens down short-term variations in plasma glucocorticoid levels and are not particularly sensitive to circadian rhythms (Dantzer *et al*., 2014; Palme 2019). This measure is non-invasive and reflects the overall concentration of plasma glucocorticoids that an individual has secreted, metabolized and excreted over a variable time window, the length of which varies between 6 to 24 h depending upon the species (Palme *et al*., 2005; Sheriff *et al*., 2011), with a mean of 12 h in roe deer (Dehnhard *et al*., 2001). Our aim was to test how baseline stress levels were associated with potential anthropogenic stressors (such as roads, houses and built-up areas), and to what extent this relationship might be modulated by behavioural adjustments in terms of use of refuge habitat over the diurnal cycle. Because human activities and infrastructure are often perceived as sources of risk for wildlife (Frid and Dill, 2002), we expected that roe deer living closer to anthropogenic structures would exhibit higher baseline stress levels. This relationship should be stronger during the day, when the human disturbance is the highest, than at night. Indeed, previous studies have shown that roe deer particularly avoid human-disturbed habitats during the day, likely due to higher perceived risk (Padié *et al*., 2015). However, roe deer may also buffer human disturbance by adjusting their spatial behaviour, notably by using woodland refuges during daytime (Bonnot *et al*. 2013; Martin *et al*., 2018). Therefore, we predicted that the negative relationship between stress levels and proximity to anthropogenic structures would be buffered as the use of refuge habitats increased, and particularly so during the day.

## 2. Material and methods

### 2.1. Study sites

This study was conducted on a population of wild roe deer living in south-western France, near Aurignac (43° 13′ N, 0° 52′ E, 10 000 ha). This population has an oceanic climate with dry summers (annual mean temperature: 11.7°C; annual mean precipitation: 794 mm; data from climate-data.org) and lives in a fragmented agricultural landscape mostly composed of open habitats, such as meadows and cultivated fields (31 and 36% of the study site, respectively), inter-mixed with small woodland patches (19%) and two main forests (14%; see Martin *et al*., 2018 for further details). The open habitats provide abundant and high-quality food resources for roe deer most of the year (Abbas *et al*., 2011), but they may be associated with potential sources of stress due to higher intensity of human activities compared to forest habitat (Bonnot *et al*., 2013). This roe deer population is not exposed to lethal or sublethal methods employed by farmers, as they do not cause significant damage to crops, but it is hunted on a regular basis during summer (June–August, bucks only) and by drive hunts with dogs during autumn–winter (September–February).

### 2.2. Field data collection

As part of a long-term capture-mark-recapture programme initiated in 1996, 6 days of capture occur each year, between the beginning of January and the beginning of March. We used large-scale drives with 30 to 100 beaters and up to 4 km of long nets. Upon capture, deer were transferred to a wooden retention box providing darkness and ventilation until the marking procedure. Roe deer were tranquilized just after capture by intramuscular injection of acepromazine (Calmivet, Vetoquinol, France; targeted dose of 0.075 mg/kg).

Marking procedures lasted ~15 min and were performed by trained handlers. Each individual was weighed (to the nearest 10 g), and sex and age were recorded. Individuals were divided into two age classes (juveniles for 8–10 month-old individuals or adults for >18 month-old individuals) based on tooth eruption patterns (Hewison *et al*., 1999). After aging, each individual was marked with two ear tags. Moreover, most were equipped with a GPS collar (Lotek 3300 GPS, Lotek Small WildCell GSM, Vectronic GPS PLUS-1C Store On Board, Vectronic GPS PLUS Mini-1C) programmed to obtain a GPS location every 6 h (at 00:00, 06:00, 12:00 and 18:00). All individuals were not equipped due the limitation of GPS collars available. All capture and marking procedures were done in accordance with French and European laws for animal welfare (prefectural order from the Toulouse Administrative Authority to capture and monitor wild roe deer, agreement no. A31113001 delivered by the Departmental Authority of Population Protection, ethical authorization by the French Government).

### 2.3. Collection and extraction of FCMs

Faecal samples were collected directly from the rectum during the marking procedure between 2012 and 2017 and held at −4°C for a maximum of 3 h before being stored at −20°C until analyses. Marking procedures took place over a period of a few hours (2:00 pm to 7:00 pm).

FCMs were extracted following a methanol-based procedure and assayed using a group-specific 11-oxoaetiocholanolone enzyme immunoassay (EIA), as previously described (Möstl *et al*., 2002) and validated for roe deer (Zbyryt *et al*., 2017). Briefly, each faecal sample was homogenized and 0.5 ± 0.005 g of homogenate was transferred to a glass tube containing 5 ml of a 80% methanol solution. The suspended samples were vortexed at 1500 rpm for 30 min and centrifuged at 2500 g for 15 min (Palme *et al*., 2013). An aliquot of the supernatant was further diluted (1:10) with assay buffer prior to EIA analysis. Measurements were carried out in duplicate (intra- and inter-assay coefficients of all samples were <10 and 15%, respectively) and the results expressed as nanograms per gram of wet faeces (ng/g). Following the suggestion of Taff *et al*. (2018), we also calculated repeatability estimates of FCMs that reflect consistent among-individual differences across time and contexts (Réale *et al*., 2007), and, therefore, measure the proportion of observed variance in FCM levels attributable to among-individual differences.

### 2.4. Home range determination and landscape variables

Home ranges were calculated using GPS data after removing the first 10 days following capture and release, since capture and handling are known to induce a transient modification of space use (Morellet *et al*., 2009). For each individual, we calculated a home range based on the 15 following days using the 90% fixed kernel method excluding locations during excursions (Worton 1989; Börger *et al*., 2006) with the ‘adehabitatHR’ R package (Calenge 2006). We hypothesized that the home range used during this particular period would be representative of the home range used just prior to capture, since roe deer exhibit high spatial fidelity (Hewison *et al*., 1998). Based on aerial photographs of the study site (from the IGN’s BD Ortho, http://professionnels.ign.fr/bdortho-50cm), we manually digitized homogeneous habitat polygons (in ArcView GIS 3.3, Esri, Redlands, CA, USA). Each polygon was then assigned to a habitat type (e.g. woodland, hedgerow, meadow or crop; the full list is available in Supplementary Data 1) from field observations each summer.

For each home range, we calculated the proportion of surface area composed of refuge habitats (woodland, hedgerows and scrubland). Here, we considered that the proportion of available refuge habitat within the home range (comprised between 0.07 and 0.99, with an average value of 0.35) reflected habitat selection when that individual established its home range within the study area (second-order habitat selection *sensu*Johnson 1980). We also calculated the probability that an individual used refuge habitat during the day (variable used in Set 1 hereafter) and at night (variable used in Set 2 hereafter), as the ratio of the number of GPS locations within a refuge habitat divided by the total number of GPS locations during daytime (at 12:00) and night-time (at 00:00) for that individual (see Bonnot *et al*., 2015 for a similar approach). During the day, this probability ranged between 0.11 and 1.00, with an average value of 0.72, whilst at night, it ranged between 0.00 and 1.00, with an average value of 0.32. This describes the selection of refuge habitat within the home range (third-order habitat selection *sensu*Johnson 1980). We considered these two scales because, so far, most studies (e.g. Jachowski *et al*., 2012) have only considered what we refer to here as available refuge habitat, whereas animals likely respond to environmental stressors at multiple spatial and temporal scales. Finally, for each individual, we calculated the average distance in meters between the GPS location and the nearest anthropogenic structure (roads, houses and other buildings) considering their locations during daytime (at 12:00), and night-time (at 00:00) separately. This distance varied from 122 to 734 m during daytime, with an average of 280 m; and from 67 to 549 m during night-time, with an average of 211 m.

### 2.5. Statistical analyses

Individual repeatability of FCMs was estimated using 261 observations of 221 individuals and mixed models using the restricted maximum likelihood (REML) method, with the ‘rptR’ package (Stoeffel *et al*., 2017) for Gaussian distributions. Individuals sampled only once were included in these analyses as this procedure may improve power to estimate among-individual variance, so avoiding biased results (Martin *et al*., 2011).

To analyze variation in FCM levels, we performed linear mixed-effects models (LMMs) on 125 observations of 116 individuals. Fewer animals were used for these analyses compared to the repeatability analyses because not all animals were equipped with GPS collars. One individual with an extremely low FCM value (<100 ng/g) was removed, because we suspected some problems with sample quality and/or possible contamination (Lexen *et al*., 2008). Two individuals with particularly high FCM values (>4500 ng/g) were also identified in our dataset. Consequently, we present results including these two values in the main text and excluding them in Supplementary Data, which did not change the main biological relationships (see Supplementary File 2). FCM level was analyzed as the response variable in four sets of models, each testing a specific hypothesis:

Sets 1 and 2: to test the relationship between proximity to anthropogenic structures and stress level and to evaluate the extent to which use of refuge habitat during daytime (Set 1) and night-time (Set 2) could influence this relationship, we included mean distance to the nearest anthropogenic structure during daytime (or night-time), probability of using refuge habitat during daytime (or night-time) and their two-way interaction. Because different types of structure might elicit different behavioural and physiological responses, we also included the type of the nearest anthropogenic structure (roads *versus* buildings) during daytime (or night-time), and the three-way interaction between mean distance to the nearest anthropogenic structure during daytime (or night-time), probability of using refuge habitat during daytime (or night-time) and the type of the nearest anthropogenic structure.

Sets 3 and 4: to test the relationship between proximity to anthropogenic structures and stress level and to evaluate the extent to which availability of refuge habitats could influence this relationship, we included mean distance to the nearest anthropogenic structure during daytime (Set 3) or night-time (Set 4), proportion of woodland patches in the home range and their two-way interaction. In addition, we included the type of the nearest anthropogenic structure (roads *versus* buildings) during daytime (or night-time), and the three-way interaction between mean distance to the nearest anthropogenic structure during daytime (or night-time), the proportion of woodland patches in the home range and the type of the nearest anthropogenic structure.

We ran the four sets of models separately because of the strong collinearity between some explanatory variables for daytime and night-time (e.g. mean distance to the nearest anthropogenic structure *r* = 0.73; *P* < 0.001) and for some landscape variables (e.g. correlation between the probability of using a refuge habitat during daytime and proportion of woodland patches in the home range *r* = 0.74; *P* < 0.001). In addition to the variables mentioned above, candidate models included age (two modalities, juveniles versus adults), sex, body mass, ambient temperature, year quality, Julian date of capture and timing of sampling (as time elapsed between sunrise and sample collection). Indeed, FCM levels can be affected by ambient temperature (Huber *et al*., 2003). Temperature was taken as the maximal ambient temperature the day prior to capture (comprised between −0.3 and 19.2°C, with an average value of 9.7°C) because of FCM indexes plasma cortisol 8 to 23 h before faeces sampling (Dehnhard *et al*., 2001). In addition, we included the population average body mass of juveniles captured the following winter (comprised between 16.4 and 17.4 kg, with an average value of 16.9 kg) to control for annual variation in resource availability and quality (see Hamel *et al*., 2009). In each set of models, body mass was standardized (using residuals from a model including the additive effects of sex and age). Individual identity was included as a random effect to avoid pseudo-replication issues (Hurlbert 1984). FCM was log-transformed to achieve normality of model residuals. To select the best models of variation in FCM level, we used a model selection procedure based on the second-order Akaike information criterion (AICc, Burnham and Anderson, 2002). Models with a difference in AICc (ΔAICc) >2 units from the best model were considered to have less support, following Burnham and Anderson (2002). In addition, we removed models within two AICc units of the top model that differed from a higher-ranking model by the addition of one or more parameters. These were rejected as uninformative, as recommended by Arnold (2010) and Richards (2008). We then applied a conditional model averaging procedure to estimate parameters. In addition, we calculated AICc weights (AICcw) to measure the relative likelihood that a given model was the best among the set of fitted models. Normality of the residuals for the selected models was tested (Shapiro–Wilk test) and visually assessed with histograms. Goodness-of-fit was assessed by calculating conditional (i.e. total variance explained by the best-supported model) and marginal (i.e. variance explained by fixed effects alone) *R*^2^ values (Table 2) and standard residual plot techniques (Nakagawa and Schielzieth, 2013). In order to interpret the results from models using space use behaviour during daytime and night-time, we also compared the average distance to the nearest anthropogenic structure between day and night locations using a paired *t*-test. All analyses were carried out with R version 3.6.0 (R Development Core Team 2016), using the lmer function from the lme4 package (Bates *et al*., 2014) and model.avg function from the MuMIn package (Barton 2016).

## 3. Results

**Figure 1:**
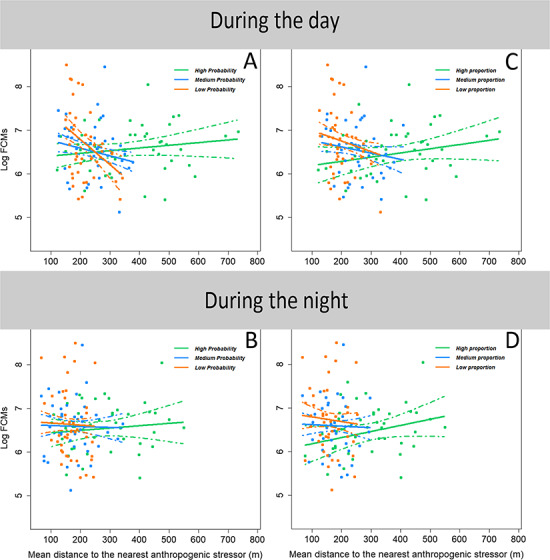
**:** Relationship between FCM level and (**A**) mean distance to the nearest anthropogenic structure during daytime in relation to the probability of using refuge habitat during daytime, (**B**) mean distance to the nearest anthropogenic structure during night-time in relation to the probability of using refuge habitat during night-time, (**C**) mean distance to the nearest anthropogenic structure during daytime in relation to the proportion of woodland in the home range, (**D**) mean distance to the nearest anthropogenic structure during night-time in relation to proportion of woodland in the home range in the roe deer population of Aurignac. Points represent observed values, lines represent model predictions and dashed lines represent the 95% confidence interval. The probability of using refuge habitat was >0.77 for the ‘high’ category (*n* = 43), between 0.65 and 0.77 (*n* = 42) for the ‘medium’ category, and <0.65 for the ‘low’ category (*n* = 42). The proportion of woodland in the home range was >0.35 for the ‘high’ category (*n* = 43), between 0.18 and 0.35 for the ‘medium’ category (*n* = 42) and <0.18 for the ‘low’ category (*n* = 42)’. The proportion might have seemed different when comparing Figs 1 and 2 (now Fig. 1A and C) because—for example, individuals that have a high probability of using refuge habitat do not necessarily have a high proportion of these refuge habitats in their home ranges, and vice versa**.** The relationship represented in Fig. 1b was not retained in the set of best models describing the data. See Table 1 for equations and statistics related to panels A and B, and Table 2 for panels C and D

In our sample (*n* = 125), FCM levels ranged from 167 to 4914 ng/g, with an average value of 934 ng/g. FCM levels were moderately repeatable: *R* = 0.28, 95% confidence interval = [0.01, 0.52], *P* value < 0.01. The model selection procedure to explain FCM variations in relation to roe deer spatial behaviour during daytime (Supplementary Data 3 and 4—daytime models) provided consistent results and included year quality, mean distance to the nearest anthropogenic structure, probability of using refuge habitat (in Set 1) or the proportion of available refuge habitat in the home range (in Set 3), as well as the two-way interaction between refuge and distance to anthropogenic structures (Tables 1 and 2, daytime). Specifically, roe deer that were closer to anthropogenic structures during daytime also exhibited higher FCM levels, but this relationship disappeared as the probability of using refuge habitat during daytime increased (Table 1; Fig. 1A) or as the proportion of refuge habitat in the home range increased (Table 2; Fig. 1C). For example, for individuals that did not use refuge habitat very frequently (probability lower than 0.65), FCM levels increased, on average, by 73% for every 100 m nearer to an anthropogenic structure, whilst they remained stable for individuals that made intensive use of refuge habitat (probability higher than 0.65). On the contrary, when there was only a low availability of refuge habitat in an individual’s home range (proportion of available refuge habitat <0.18), FCM levels increased by only 20% for every 100 m nearer an anthropogenic structure, whilst they remained stable when the availability of refuge habitat was higher (proportion higher than 0.65). In addition, FCM levels increased as the quality of the year decreased such that FCM levels in the poorest year of study were, on average, 36% higher than those in the best year (Supplementary Data 5).

**Table 1:** **:** Characteristics of the selected linear mixed-effect models for explaining variation in FCM levels in the roe deer population of Aurignac in relation to use of refuge habitat and proximity to anthropogenic infrastructure during daytime and night-time

**Parameter**	**Estimate**	**CI**
**Daytime Set 1** **(** *R* ^2m^:0.15; *R*^2c^:0.41)		
Intercept	15.606	9.534 to 21.664
Distance to human infrastructure	−0.011	−0.016 to −0.005
Probability of using refuge habitat	−2.888	−4.293 to −1.480
Year quality	−0.383	−0.741 to −0.025
Distance to human infrastructure * probability of using refuge habitat	0.011	0.006 to 0.017
**Night-time Set 2** (*R*^2m^:0.06; *R*^2c^:0.40)		
Intercept	14.139	7.905 to 20.373
Temperature	−0.024	−0.053 to 0.005
Year quality	−0.438	−0.806 to −0.070

The effect of mean distance to the nearest anthropogenic structure during daytime (distance to human infrastructure), probability of using refuge habitat during daytime, maximal temperature the day before capture (temperature), year quality (indexed by the population average body mass of juveniles captured during the following winter) and the two-way interaction between mean distance to the nearest anthropogenic structure and probability of using refuge habitats during daytime were fitted. Models included individual identity and year of capture as random effects. *R*^2m^ and *R*^2c^ are the marginal and conditional explained variance of the models, respectively. CI stands for confidence interval. See text for the definition of model sets

**Table 2:** Characteristics of the selected linear mixed-effect models for explaining variation in FCM levels in the roe deer population of Aurignac in relation to available refuge habitat and proximity to anthropogenic infrastructure during daytime and night-time

**Parameter**	**Estimate**	**CI**
**Daytime Set 3** **(** *R* ^2m^:0.11; *R*^2c^:0.38)		
Intercept	15.463	9.211 to 21.714
Distance to human infrastructure	−0.003	−0.006 to −0.001
Proportion of woodland patches	−2.421	−3.937 to −0.902
Year quality	−0.468	−0.837 to −0.098
Distance to human infrastructure * proportion of woodland patches	0.007	0.003 to 0.012
**Night-time Set 4** (*R*^2m^:0.11; *R*^2c^:0.45)		
Intercept	14.435	8.199 to 20.672
Distance to human infrastructure	−0.002	−0.005 to 0.001
Proportion of woodland patches	−1.896	−3.281 to −0.510
Temperature	−0.024	−0.053 to 0.005
Year quality	−0.446	−0.813 to −0.080
Distance to human infrastructure * proportion of woodland patches	0.005	0.001 to 0.010

The effect of mean distance to the nearest anthropogenic structure during daytime (distance to human infrastructure), proportion of woodland patches in the home range, maximal temperature the day before capture (temperature), year quality (indexed by the population average body mass of juveniles captured during the following winter) and the two-way interaction between mean distance to the nearest anthropogenic structure during daytime and proportion of woodland patches in the home range were fitted. Models included individual identity and year of capture as random effects. *R*^2m^ and *R*^2c^ are the marginal and conditional explained variance of the model, respectively. CI stands for confidence interval. See text for the definition of model sets

Both models selected to explain FCM variations in relation to night-time spatial behaviour (Supplementary Data 3 and 4—night-time models) included the effects of year quality and maximum ambient temperature the day prior to capture (Tables 1 and 2 night-time), with the highest FCM levels observed with lower maximum ambient temperature (Supplementary Data 5). In contrast with daytime, neither the main effects of mean distance to the nearest anthropogenic structure and the probability of using refuge habitats nor their two-way interaction (Set 2) was retained in the best model. In contrast, mean distance to the nearest anthropogenic structure, proportion of refuge habitat in the home range and their two-way interaction (Set 4) still featured in the set of best models. However, the relationship was particularly weak compared to daytime spatial behaviour (Table 2), such that FCM levels increased, on average, by only 6% for every 100 m nearer to an anthropogenic structure at night. Furthermore, this interaction did not feature in the set of best models when removing the two individuals with very high FCM values (Supplementary Data 2).

Finally, our results showed that, on average, individuals ranged 68 m closer to anthropogenic structures during night-time compared to daytime (*t* = 9.10; df = 124; *P* < 0.001; standard error = 7.60).

## 4. Discussion

The aims of this study were 2-fold. First, we explored the relationship between sources of anthropogenic disturbance and baseline stress levels in wild roe deer, whilst taking into account potential effects of ambient temperature, date of capture, timing of sampling, age, sex, body mass and resource availability. Second, we determined the extent to which spatio-temporal adjustments of behaviour could contribute to buffer a negative relationship between anthropogenic disturbance and baseline stress levels. Similarly to Shutt *et al*. (2014), our results show that exposure to anthropogenic disturbance is not always associated with higher stress levels in a wild mammal. In particular, proximity to anthropogenic structures at night was unrelated (or only weakly related) to FCM levels, whilst the availability and use of refuge habitat during the day attenuated the negative relationship between these stressors and FCM levels.

The average level of observed FCMs is similar to those reported by Zbyryt *et al*. (2017) in two roe deer populations exposed to human disturbance, but free from predators (mean FCM levels of 874 ng/g). The level of repeatability in FCMs that we found is also consistent with a recent meta-analysis that estimated an overall repeatability of *R* ~0.29 for glucocorticoid levels (Taff *et al*., 2018). Consistent with previous studies on other species (Arlettaz *et al*., 2007 on black grouse *Tetrao tetrix*; Bourbonnais *et al*., 2013 on brown bear *U. arctos*; Rehnus *et al*., 2014 on mountain hare *Lepus timidus*), our results showed that FCM levels increased as roe deer ranged closer to anthropogenic structures. It has previously been suggested that habituation could occur in environments that are regularly disturbed by human activities (Walker *et al*., 2006; Cyr and Romero, 2009; Bonnot *et al*., 2018), which could also potentially be the case in our study. However, we found a link between FCMs and exposure to anthropogenic disturbance only under certain conditions, suggesting a plastic response mediated by roe deer behaviour. In particular, an individual was more likely to be stressed by proximity to anthropogenic structures when it used open habitats during the day, when it did not make extensive use of refuge habitats, or when refuges were not widely available. Previous studies on the same roe deer population have shown that roe deer modified their spatial behaviour when in proximity with anthropogenic structures (Coulon *et al*., 2008), restricted their routine movement to safe habitats during daytime and the hunting season (Martin *et al*., 2018) but also decreased their use of risky habitat and reduced their distance to cover when risk increased (Padié *et al*., 2015). We were able to demonstrate that the protective influence of wooded refuge habitats resulted in attenuated stress levels, indicating that these behavioural adjustments could mitigate stress levels from a physiological point of view. This suggests that the physiological effects of human disturbance on wild mammals may be more complex than previously assumed (Dantzer *et al*., 2014) and may not only depend on the spatial distribution of human disturbance but also on the individual’s ability to access and use refuge habitat when the disturbance is high.

Whilst we observed strong associations between spatial behaviour and FCM levels, we did not establish a causal relationship. Therefore, we cannot exclude the possibility that our results reflect the influence of glucocorticoid levels on spatial behaviour. Indeed, several studies (e.g. Raoult *et al*., 2012), including in humans (e.g. Cueva *et al*., 2015), have shown that high glucocorticoid levels could promote risky behaviours and boldness. To our knowledge, this phenomenon has never been formally demonstrated in wild large herbivores but could also explain, at least in part, the observed relationship between distance to anthropogenic disturbance and FCM levels.

The observation that proximity to anthropogenic structures at night was unrelated (or only weakly related) to an individual’s stress level suggests that darkness alters the perception of risk in roe deer. This suggests that night-time could also provide a form of refuge from perceived stressors for wild animals, as suggested by the recent global study of Gaynor *et al*. (2018) but also that night could facilitate habituation to stressors. Again, this result is in accordance with previous work on the same roe deer population that showed that roe deer adjusted their spatial behaviour, using more woodland habitat during daytime when proximity to human activity was high, whilst there was no temporal difference in spatial behaviour in deer occupying woodland far from human activity (Bonnot *et al*., 2013). Indeed, large herbivores face a trade-off between acquisition of high-quality food resources and exposure to anthropogenic stressors (Bonnot *et al*., 2015). Whilst open habitats are often associated with high levels of disturbance, forage quality is often higher (Abbas *et al*., 2011, Hewison *et al*., 2009) such that juveniles in these habitats weigh up to 3 kg more than their counterparts in forested habitat (Hewison *et al*., 2009), which can have implications for individual fitness (Hewison *et al*., 2002). Whilst it is widely reported that many wild animals have increased their level of nocturnal activity in the face of human disturbance (Gaynor *et al*., 2018), we are not aware of any study to date that has investigated the relationship between FCM levels and spatial behaviour in relation to the degree of nocturnality. Our results suggest that the cost of exploiting open habitats in terms of exposure to stress is lower during night-time compared to daytime. Consequently, animals may mitigate costs associated with proximity to humans by using open habitat during the night.

Overall, our biological relationships are similar, whether considering use or availability of refuge habitat. However, the magnitude of the relationships differs. We found a stronger relationship when considering the use of refuge habitat (Fig. 1A), compared to its availability (Fig. 1C). This difference may be related to among-individual differences in space use (Spiegel *et al*., 2017): some individuals may consistently use more anthropogenic areas, whilst others may consistently use more refuge habitat, and this, regardless of the availability of these habitats. Therefore, accounting for how individuals actually use their space may prove more informative than simply considering spatial availability of particular features when examining the relationships between FCMs, human activities and landscape structure.

Overall, our results suggest that the stress levels of wild ungulates are strongly influenced by proximity to human activities, but that these effects are buffered by both spatial and temporal behavioural adjustments. From an evolutionary point of view, we can, therefore, expect wild animals in human-disturbed habitats to exhibit temporal shifts in their spatial behaviour, with an increase in the nocturnal use of habitats that appear risky during the day. In terms of locally applicable management and conservation strategies for free-ranging animals, preservation or restoration of patches of refuge habitat in proximity to human infrastructure could help to mitigate stress levels and the potential negative consequences on health, fitness and, ultimately, population dynamics of living in the Anthropocene.

## Funding

This project was funded by INRA, VetAgro Sup and ONCFS; was supported by the ‘Mov-It’ ANR grant (ANR-16-CE02-0010-02) to A.J.M.H. and N.M.; and was also performed in the framework of the LABEX ECOFECT (ANR-11-LABX-0048) of Université de Lyon, within the programme ‘Investissements d’Avenir’ (ANR-11-IDEX-0007).

## Supplementary Material

Supplementary_materials_coaa086
