# Supplementary material for: Under cover of the night: context-dependency of anthropogenic disturbance on stress levels of wild roe deer Capreolus capreolus
Source: Conserv Physiol. 2020 Sep 22;8(1):coaa086. doi: 10.1093/conphys/coaa086 (PMC7507870; doi:10.1093/conphys/coaa086)
Supplement: Supplementary_materials_coaa086 [file supplementary_materials_coaa086.zip › ESM_1 Full list GIS.docx]

**Under cover of the night: context-dependency of anthropogenic disturbance on stress levels of wild roe deer *Capreolus capreolus***

Jeffrey Carbillet^1,2,*^, Benjamin Rey^3^, Rupert Palme^4^, Nicolas Morellet^1^, Nadège Bonnot^5^, A.J.M. Hewison^1^, Yannick Chaval^1^, Bruno Cargnelutti^1^, Emmanuelle Gilot-Fromont^2,3^, Hélène Verheyden^1^

^1 Université de Toulouse, INRAE, CEFS, F-31326, Castanet Tolosan, France^

^2 Université de Lyon, VetAgro Sup, Campus vétérinaire de Lyon, F-69280 Marcy-l’Etoile, France^

^3 Université de Lyon, Université Lyon 1, CNRS, Laboratoire de Biométrie et Biologie Evolutive UMR 5558, F-69622 Villeurbanne, France^

^4 Unit of Physiology, Pathophysiology, and Experimental Endocrinology, Department of Biomedical Sciences, University of Veterinary Medicine, Vienna, 1210, Austria^

^5 INRAE, EFNO, F-45290, Nogent-sur-Vernisson, France^

^* Corresponding author: Tel: +335 61 28 51 32 Email:^ [^jeffrey.cm@live.fr^](mailto:jeffrey.cm@live.fr)

**Supplementary data 1**:

Full list of habitat types used to assign each polygon to a habitat type from field observations each summer.

- - - - - **Cereal**: wheat, barley, corn, oats, wheat + barley + oats, barley + oats, other cereal
        - **Cereal + rape**
        - **Cereal + meadow***: barley* + rye-grass
        - **Rape**
        - **Water**: reservoir, pond, lake, river
        - **Enclosure**
        - **Wilderness**: sector colonised by ligneous species
        - **Hedgerow**
        - **Uncultivated land**
        - **Garden**
        - **Leguminous plants**: pea, field bean and other leguminous plants
        - **Lucerne**
        - **Strips cover with grass**
        - **Woodland**: forest, grove, poplar grove.

Grove correspond to every non-linear parcel or parcel with a width higher than 25 meters.

- - - - - **Market gardening**
        - **Tree filled parks**: private parcels filled with scattered trees close to a building.
        - **Lawn**
        - **Natural meadow**
        - **Artificial meadow**: rye-grass, Lucerne + meadow, rye-grass + clover, clover
        - **Roads**: road D90, private roads, road D81e, municipal road, road D17, road D5, road D635, road D3, road D36, road D52, road D81, road D17e, road D78, road D98, road D75, road D98, road D81b, road D75d, road D8, road D81d, road D635, road D8d, road D8, road D6b, road D96a, road D96d, road D96b, road D96, road D36d, road D48d, road D48, road D96c, road D6h, road D13, road D96c, road D75e, road D78b, road D90d, road D3d, road D23, road D84, road D23a, road D3b, road D3a, road D90b, road D84d, road D48a, road D36b, road D48c, road D36c, road D52b, road D36a, road D6a, road D48b, road D23e, road D90, road D90a, road D81e, road D75k, road D81c, road D98d, road D5f, road D52f, road D6, road D98
        - **Path**: private and municipal path
        - **Construction site**
        - **Buildings**: houses, depot, swimming pool, water tower, rubbish tip, ruin, tennis courts, sawmill, doghouse, cimetery
        - **Car park**
        - **Farm annexes**
        - **Soya**
        - **Sorghum**
        - **Sunflower**
        - **Orchard**
        - **Vine**
