# Supplementary material for: Under cover of the night: context-dependency of anthropogenic disturbance on stress levels of wild roe deer Capreolus capreolus
Source: Conserv Physiol. 2020 Sep 22;8(1):coaa086. doi: 10.1093/conphys/coaa086 (PMC7507870; doi:10.1093/conphys/coaa086)
Supplement: Supplementary_materials_coaa086 [file supplementary_materials_coaa086.zip › ESM_2 (without 2 high points).docx]

**Under cover of the night: context-dependency of anthropogenic disturbance on stress levels of wild roe deer *Capreolus capreolus***

Jeffrey Carbillet^1,2,*^, Benjamin Rey^3^, Rupert Palme^4^, Nicolas Morellet^1^, Nadège Bonnot^5^, A.J.M. Hewison^1^, Yannick Chaval^1^, Bruno Cargnelutti^1^, Emmanuelle Gilot-Fromont^2,3^, Hélène Verheyden^1^

^1 Université de Toulouse, INRAE, CEFS, F-31326, Castanet Tolosan, France^

^2 Université de Lyon, VetAgro Sup, Campus vétérinaire de Lyon, F-69280 Marcy-l’Etoile, France^

^3 Université de Lyon, Université Lyon 1, CNRS, Laboratoire de Biométrie et Biologie Evolutive UMR 5558, F-69622 Villeurbanne, France^

^4 Unit of Physiology, Pathophysiology, and Experimental Endocrinology, Department of Biomedical Sciences, University of Veterinary Medicine, Vienna, 1210, Austria^

^5 INRAE, EFNO, F-45290, Nogent-sur-Vernisson, France^

^* Corresponding author: Tel: +335 61 28 51 32 Email:^ [^jeffrey.cm@live.fr^](mailto:jeffrey.cm@live.fr)

**Supplementary data 2**: Analyses without two high values of FCMs (> 4 500 ng/g)

**I) Use of refuge habitats**

Performance of the subset of candidate linear mixed-effect models within a ΔAICc < 2 fitted to investigate variation in faecal glucocorticoids metabolite levels in the roe deer population of Aurignac according to daytime (A) and night-time (B) use of refuge habitats. Model(s) in bold was/were used for estimation of parameters, and averaged when more than one model was considered after removing models that differed from a higher-ranking model by the addition of one or more parameters. These were rejected as uninformative, as recommended by Arnold (2010) and Richards (2008). Our set of candidate models was composed of all simpler models that included sex, age, year quality, body mass, Julian date of capture (Date), sampling timing (time elapsed between sunrise and sample collection), mean distance to the nearest anthropogenic structure (Anthropogenic distance) during daytime (A) and night-time (B), probability to use refuge habitats during daytime (PRHD) or night-time (PRHN), maximal temperature the day before capture (Temperature), type of the nearest anthropogenic structure during daytime (StructureD) or night-time (StructureN), and the three-way interaction between mean distance to the nearest anthropogenic structure, probability to use refuge habitats during daytime (A) and nighttime (B), and type of the nearest anthropogenic structure during daytime (StructureD) or night-time (StructureN). Individual identity was included as a random effect. AICc is the value of the corrected Akaike’s Information Criterion and K is the number of estimated parameters for each model. The ranking of the models is based on the differences in the values for ΔAICc and on the Akaike weights (AICw).

A) Daytime model

| **Models** | **K** | **AICc** | **ΔAICc** | **AICw** |
| --- | --- | --- | --- | --- |
|  | | | | |
| **Anthropogenic distance+Year quality+PRHD+**  **Anthropogenic distance*PRHD+Temperature** | **8** | **224.9** | **0.00** | **0.45** |
| Anthropogenic distance+Year quality+PRHD+  Anthropogenic distance*PRHD+Temperature+Body mass | 9 | 226.6 | 1.67 | 0.19 |
| Anthropogenic distance+Year quality+PRHD+  Anthropogenic distance*PRHD+Temperature+Age | 9 | 226.6 | 1.72 | 0.19 |
| Anthropogenic distance+Year quality+PRHD+  Anthropogenic distance*PRHD+Temperature+Sex | 8 | 226.8 | 1.94 | 0.17 |

B) Night-time model

| **Models** | **K** | **AICc** | **ΔAICc** | **AICw** |
| --- | --- | --- | --- | --- |
|  | | | | |
| **Year quality+Temperature** | **5** | **231.7** | **0.00** | **0.27** |
| Year quality+Temperature+StructureN | 6 | 232.6 | 0.95 | 0.17 |
| Year quality+Temperature+Body mass | 6 | 233.0 | 1.39 | 0.13 |
| Year quality+Temperature+Age | 6 | 233.3 | 1.63 | 0.12 |
| Year quality+Temperature+Sampling timing | 6 | 233.3 | 1.69 | 0.11 |
| Year quality+Temperature+Sex | 6 | 233.5 | 1.86 | 0.11 |
| Year quality+Temperature+Date | 6 | 233.6 | 1.93 | 0.10 |

| **Parameter** | **Estimate** | **CI** |
| --- | --- | --- |
| **Daytime set 1**  **(**R^2m^:0.15 ; R^2c^:0.39) |  |  |
| Intercept | 15.313 | 9.570 to 21.042 |
| Distance to human infrastructure | -0.010 | -0.015 to -0.005 |
| Probability of using refuge habitat | -2.701 | -4.073 to -1.326 |
| Year quality | -0.376 | -0.714 to -0.038 |
| Distance to human infrastructure * Probability of using refuge habitat | 0.011 | 0.005 to 0.017 |
| **Night-time set 2**  **(**R^2m^:0.08 ; R^2c^:0.35) |  |  |
| Intercept | 14.127 | 8.301 to 19.919 |
| Temperature | -0.033 | -0.060 to -0.005 |
| Year quality | -0.428 | -0.770 to -0.084 |

**Table 1** Characteristics of the selected linear mixed-effect models for explaining variation in FCM levels in the roe deer population of Aurignac in relation to use of refuge habitat and proximity to anthropogenic infrastructure during daytime and night-time. The effect of mean distance to the nearest anthropogenic structure during daytime (Distance to human infrastructure), probability of using refuge habitat during daytime, maximal temperature the day before capture (Temperature), year quality (indexed by the population average body mass of juveniles captured during the following winter), and the two-way interaction between mean distance to the nearest anthropogenic structure and probability of using refuge habitats during daytime were fitted. Models included individual identity and year of capture as random effects. R^2m^ and R^2c^ are the marginal and conditional explained variance of the models, respectively. CI stands for Confidence Interval. See text for definition of model sets.

**II) Availability of refuge habitats**

Performance of the subset of candidate linear mixed-effect models within a ΔAICc < 2 fitted to investigate variation in faecal glucocorticoids metabolite levels in the roe deer population of Aurignac according to daytime (A) and nighttime (B) space use behaviour and available refuge habitats in the home range. Model(s) in bold was/were used for estimation of parameters, and averaged when more than one model was considered after removing models that differed from a higher-ranking model by the addition of one or more parameters. These were rejected as uninformative, as recommended by Arnold (2010) and Richards (2008). Our set of candidate models was composed of all simpler models that included sex, age, year quality, body mass, Julian date of capture (Date), sampling timing (time elapsed between sunrise and sample collection), mean distance to the nearest anthropogenic structure (Anthropogenic distance) during daytime (A) and night-time (B), proportion of woodland patches in the home range (Woodland), maximal temperature the day before capture (Temperature), type of the nearest anthropogenic structure during daytime (StructureD) or night-time (StructureN), and the three-way interaction between mean distance to the nearest anthropogenic structure, proportion of woodland patches in the home range, and type of the nearest anthropogenic structure during daytime (StructureD) or night-time (StructureN). Individual identity was included as a random effect. AICc is the value of the corrected Akaike’s Information Criterion and K is the number of estimated parameters for each model. The ranking of the models is based on the differences in the values for ΔAICc and on the Akaike weights (AICw).

A) Daytime model

| **Models** | **K** | **AICc** | **ΔAICc** | **AICw** |
| --- | --- | --- | --- | --- |
|  | | | | |
| **Anthropogenic distance+Year quality+Woodland+**  **Anthropogenic distance*Woodland+Temperature** | **8** | **229.7** | **0.00** | **0.33** |
| Anthropogenic distance+Year quality+Woodland+  Anthropogenic distance*Woodland+Temperature+Age | 9 | 231.2 | 1.50 | 0.16 |
| Anthropogenic distance+Year quality+Woodland+  Anthropogenic distance*Woodland+Temperature+Body mass | 9 | 231.5 | 1.77 | 0.14 |
| **Anthropogenic distance+Year quality+Woodland+**  **Anthropogenic distance*Woodland** | **7** | **231.6** | **1.87** | **0.13** |
| **Year quality+Temperature** | **5** | **231.7** | **1.95** | **0.12** |
| Anthropogenic distance+Year quality+Woodland+  Anthropogenic distance*Woodland+Temperature+Sex | 9 | 231.7 | 1.97 | 0.12 |

B) Night-time model

| **Models** | **K** | **AICc** | **ΔAICc** | **AICw** |
| --- | --- | --- | --- | --- |
|  | | | | |
| **Year quality+Temperature** | **5** | **231.7** | **0.00** | **0.19** |
| Year quality+Temperature+Anthropogenic distance+Woodland+Anthropogenic distance*Woodland | 8 | 232.2 | 0.54 | 0.14 |
| Year quality+Temperature+StructureN | 6 | 232.6 | 0.95 | 0.12 |
| Year quality+Temperature+Body mass | 6 | 233.0 | 1.39 | 0.09 |
| Year quality+Temperature+Age | 6 | 233.3 | 1.63 | 0.08 |
| Year quality+Temperature+Sampling timing | 6 | 233.3 | 1.69 | 0.08 |
| Year quality+Temperature+Woodland | 6 | 233.5 | 1.83 | 0.08 |
| Year quality+Temperature+Sex | 6 | 233.5 | 1.86 | 0.07 |
| Year quality+Temperature+Anthropogenic distance+Woodland+Anthropogenic distance*Woodland+StructureN | 9 | 233.6 | 1.91 | 0.07 |
| Year quality+Temperature+Date | 6 | 233.6 | 1.93 | 0.07 |

| **Parameter** | **Estimate** | **CI** |
| --- | --- | --- |
| **Daytime set 3**  **(**R^2m^:0.12 ; R^2c^:0.36) |  |  |
| Intercept | 14.625 | 8.693 to 20.557 |
| Distance to human infrastructure | -0.003 | -0.006 to -0.001 |
| Proportion of woodland patches | -2.233 | -3.666 to -0.799 |
| Year quality | -0.440 | -0.787 to -0.094 |
| Temperature | -0.033 | -0.060 to -0.005 |
| Distance to human infrastructure * proportion of woodland patches | 0.007 | 0.003 to 0.011 |
| **Night-time set 4**  **(**R^2m^:0.08 ; R^2c^:0.35) |  |  |
| Intercept | 14.127 | 8.301 to 19.919 |
| Temperature | -0.033 | -0.060 to -0.005 |
| Year quality | -0.428 | -0.770 to -0.084 |

**Table 2** Characteristics of the selected linear mixed-effect models for explaining variation in FCM levels in the roe deer population of Aurignac in relation to available refuge habitat and proximity to anthropogenic infrastructure during daytime and night-time. The effect of mean distance to the nearest anthropogenic structure during daytime (Distance to human infrastructure), proportion of woodland patches in the home range, maximal temperature the day before capture (Temperature), year quality (indexed by the population average body mass of juveniles captured during the following winter), and the two-way interaction between mean distance to the nearest anthropogenic structure during daytime and proportion of woodland patches in the home range were fitted. Models included individual identity and year of capture as random effects. R^2m^ and R^2c^ are the marginal and conditional explained variance of the model, respectively. CI stands for Confidence Interval. See text for definition of model sets.


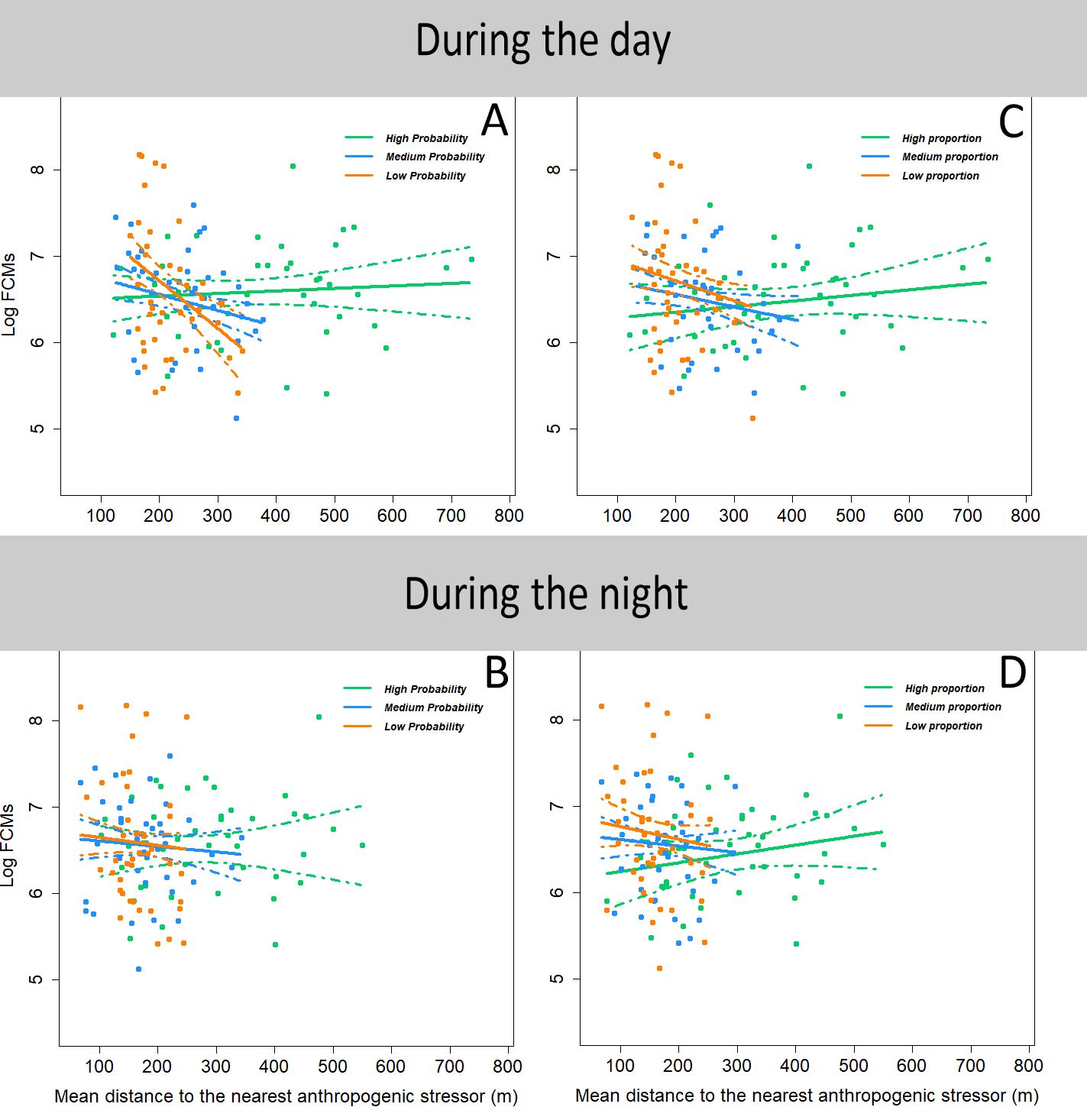


**Fig. 1** Relationship between FCMs level and; A) mean distance to the nearest anthropogenic structure during daytime in relation to the probability of using refuge habitat during daytime; B) mean distance to the nearest anthropogenic structure during night-time in relation to the probability of using refuge habitat during night-time; C) mean distance to the nearest anthropogenic structure during daytime in relation to proportion of woodland in the home range; D) mean distance to the nearest anthropogenic structure during night-time in relation to proportion of woodland in the home range in the roe deer population of Aurignac. Points represent observed values, lines represent model predictions and dashed lines represent the 95% confidence interval. The probability of using refuge habitat was > 0.77 for the “High” category (n=43), between 0.65 and 0.77 (n=42) for the “Medium” category, and < 0.65 for the “Low” category (n=42). The proportion of woodland in the home range was > 0.35 for the “High” category (n=43), between 0.18 and 0.35 for the “Medium” category (n=42), and < 0.18 for the “Low” category (n=42)”. The relationship represented in figure 1.B and 1.D were not retained in the set of best models describing the data. See table 1 for equations and statistics related to figure A & B, and table 2 for figure C & D.
