# Supplementary material for: Under cover of the night: context-dependency of anthropogenic disturbance on stress levels of wild roe deer Capreolus capreolus
Source: Conserv Physiol. 2020 Sep 22;8(1):coaa086. doi: 10.1093/conphys/coaa086 (PMC7507870; doi:10.1093/conphys/coaa086)
Supplement: Supplementary_materials_coaa086 [file supplementary_materials_coaa086.zip › ESM_3 PRHD models_revised.docx]

**Under cover of the night: context-dependency of anthropogenic disturbance on stress levels of wild roe deer *Capreolus capreolus***

Jeffrey Carbillet^1,2,*^, Benjamin Rey^3^, Rupert Palme^4^, Nicolas Morellet^1^, Nadège Bonnot^5^, A.J.M. Hewison^1^, Yannick Chaval^1^, Bruno Cargnelutti^1^, Emmanuelle Gilot-Fromont^2,3^, Hélène Verheyden^1^

^1 Université de Toulouse, INRAE, CEFS, F-31326, Castanet Tolosan, France^

^2 Université de Lyon, VetAgro Sup, Campus vétérinaire de Lyon, F-69280 Marcy-l’Etoile, France^

^3 Université de Lyon, Université Lyon 1, CNRS, Laboratoire de Biométrie et Biologie Evolutive UMR 5558, F-69622 Villeurbanne, France^

^4 Unit of Physiology, Pathophysiology, and Experimental Endocrinology, Department of Biomedical Sciences, University of Veterinary Medicine, Vienna, 1210, Austria^

^5 INRAE, EFNO, F-45290, Nogent-sur-Vernisson, France^

^* Corresponding author: Tel: +335 61 28 51 32 Email:^ [^jeffrey.cm@live.fr^](mailto:jeffrey.cm@live.fr)

**Supplementary data 3**: Performance of the subset of candidate linear mixed-effect models within a ΔAICc < 2 fitted to investigate variation in faecal glucocorticoids metabolite levels in the roe deer population of Aurignac according to daytime (A) and night-time (B) use of refuge habitats. Model(s) in bold was/were used for estimation of parameters, and averaged when more than one model was considered after removing models that differed from a higher-ranking model by the addition of one or more parameters. These were rejected as uninformative, as recommended by Arnold (2010) and Richards (2008). Our set of candidate models was composed of all simpler models that included sex, age, year quality, body mass, Julian date of capture (Date), sampling timing (time elapsed between sunrise and sample collection), mean distance to the nearest anthropogenic structure (Anthropogenic distance) during daytime (A) and night-time (B), probability to use refuge habitats during daytime (PRHD) or night-time (PRHN), maximal temperature the day before capture (Temperature), type of the nearest anthropogenic structure during daytime (StructureD) or night-time (StructureN), and the three-way interaction between mean distance to the nearest anthropogenic structure, probability to use refuge habitats during daytime (A) and nighttime (B), and type of the nearest anthropogenic structure during daytime (StructureD) or night-time (StructureN). Individual identity was included as a random effect. AICc is the value of the corrected Akaike’s Information Criterion and K is the number of estimated parameters for each model. The ranking of the models is based on the differences in the values for ΔAICc and on the Akaike weights (AICw).

A) Daytime model

| **Models** | **K** | **AICc** | **ΔAICc** | **AICw** |
| --- | --- | --- | --- | --- |
|  | | | | |
| **Anthropogenic distance+Year quality+PRHD+**  **Anthropogenic distance*PRHD** | **7** | **245.1** | **0.00** | **0.247** |
| Anthropogenic distance+Year quality+PRHD+Temperature+  Anthropogenic distance*PRHD | 8 | 245.5 | 0.35 | 0.207 |
| Anthropogenic distance+Year quality+PRHD+Temperature+  Anthropogenic distance*PRHD+Age | 9 | 246.5 | 1.34 | 0.126 |
| Anthropogenic distance+Year quality+PRHD+  Anthropogenic distance*PRHD+Body mass | 8 | 246.6 | 1.44 | 0.120 |
| Anthropogenic distance+Year quality+PRHD+  Anthropogenic distance*PRHD+Age | 8 | 246.7 | 1.56 | 0.113 |
| Anthropogenic distance+Year quality+PRHD+Temperature  Anthropogenic distance*PRHD+Body mass | 9 | 247.1 | 1.93 | 0.094 |
| Anthropogenic distance+Year quality+PRHD+  Anthropogenic distance*PRHD+Julian date | 8 | 247.1 | 1.96 | 0.093 |

B) Night-time model

| **Models** | **K** | **AICc** | **ΔAICc** | **AICw** |
| --- | --- | --- | --- | --- |
|  | | | | |
| **Year quality+Temperature** | **5** | **253.7** | **0.00** | **0.12** |
| Year quality+Temperature+StructureN | 6 | 253.7 | 0.02 | 0.12 |
| **Year quality** | **4** | **254.1** | **0.41** | **0.10** |
| Year quality+StructureN | 5 | 254.3 | 0.63 | 0.09 |
| Year quality+Temperature+Age | 6 | 254.3 | 0.65 | 0.09 |
| Year quality+Temperature+StructureN+Age | 7 | 254.4 | 0.77 | 0.08 |
| Year quality+Temperature+StructureN+PRHN | 7 | 255.1 | 1.45 | 0.06 |
| Year quality+Temperature+StructureN+Body mass | 7 | 255.1 | 1.48 | 0.06 |
| Year quality+Temperature+Body mass | 6 | 255.1 | 1.49 | 0.06 |
| Year quality+Age | 5 | 255.3 | 1.63 | 0.05 |
| Year quality+Body mass | 5 | 255.4 | 1.76 | 0.05 |
| Year quality+Temperature+PRHN | 6 | 255.5 | 1.87 | 0.05 |
| Year quality+StructureN+Body mass | 6 | 255.6 | 1.94 | 0.04 |
| Year quality+StructureN+Age | 6 | 255.6 | 1.97 | 0.04 |
