# Supplementary material for: Under cover of the night: context-dependency of anthropogenic disturbance on stress levels of wild roe deer Capreolus capreolus
Source: Conserv Physiol. 2020 Sep 22;8(1):coaa086. doi: 10.1093/conphys/coaa086 (PMC7507870; doi:10.1093/conphys/coaa086)
Supplement: Supplementary_materials_coaa086 [file supplementary_materials_coaa086.zip › ESM_5 Other effects.docx]

**Under cover of the night: context-dependency of anthropogenic disturbance on stress levels of wild roe deer *Capreolus capreolus***

Jeffrey Carbillet^1,2,*^, Benjamin Rey^3^, Rupert Palme^4^, Nicolas Morellet^1^, Nadège Bonnot^5^, A.J.M. Hewison^1^, Yannick Chaval^1^, Bruno Cargnelutti^1^, Emmanuelle Gilot-Fromont^2,3^, Hélène Verheyden^1^

^1 Université de Toulouse, INRAE, CEFS, F-31326, Castanet Tolosan, France^

^2 Université de Lyon, VetAgro Sup, Campus vétérinaire de Lyon, F-69280 Marcy-l’Etoile, France^

^3 Université de Lyon, Université Lyon 1, CNRS, Laboratoire de Biométrie et Biologie Evolutive UMR 5558, F-69622 Villeurbanne, France^

^4 Unit of Physiology, Pathophysiology, and Experimental Endocrinology, Department of Biomedical Sciences, University of Veterinary Medicine, Vienna, 1210, Austria^

^5 INRAE, EFNO, F-45290, Nogent-sur-Vernisson, France^

^* Corresponding author: Tel: +335 61 28 51 32 Email:^ [^jeffrey.cm@live.fr^](mailto:jeffrey.cm@live.fr)

**Supplementary data 5**:


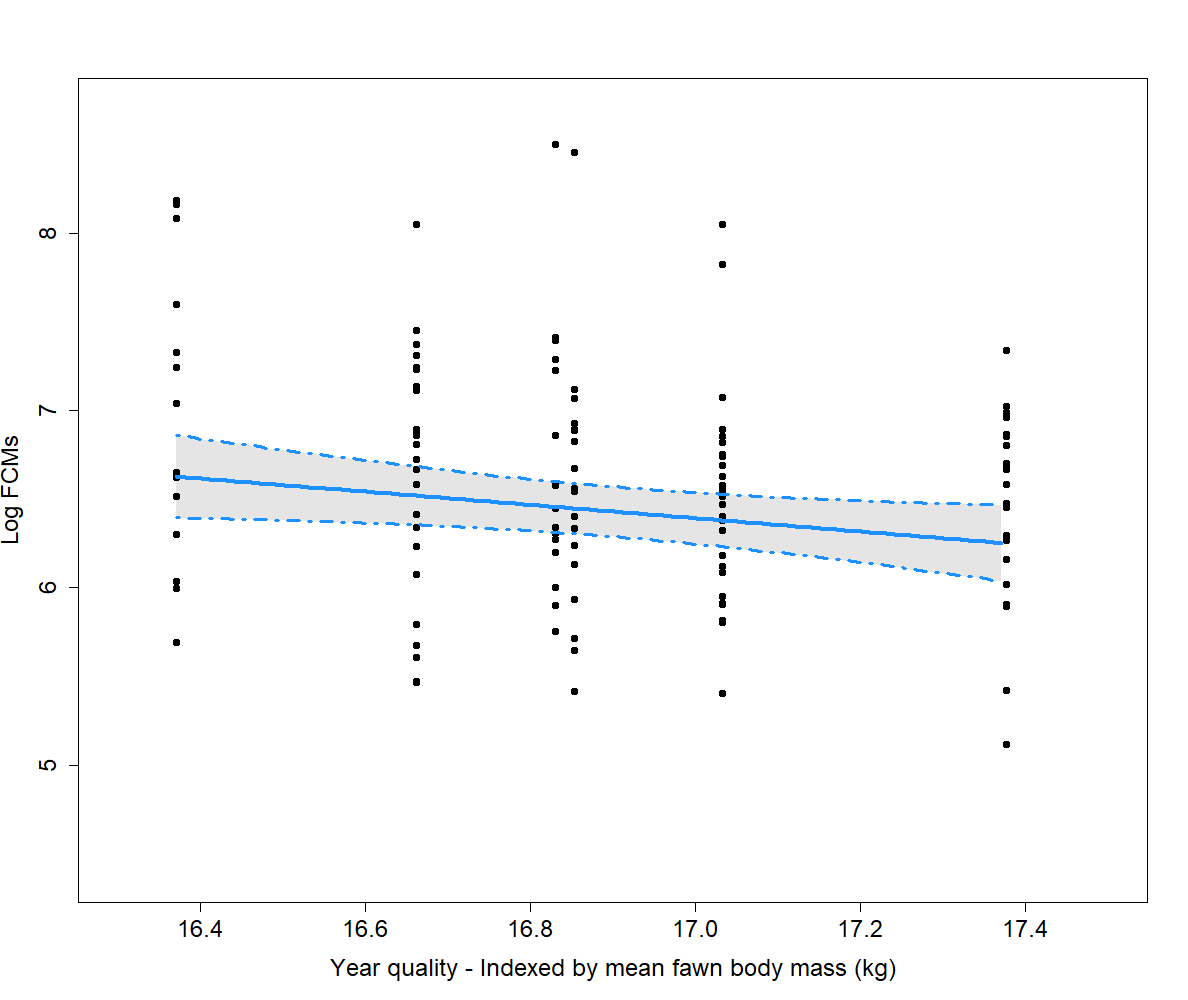


**Fig. 1** Relationship between FCMs level and year quality in the roe deer population of Aurignac. Year quality was indexed using the population average body mass of juveniles captured during the following winter. Points represent observed values, lines represent model predictions and dashed lines represent the 95% confidence interval.


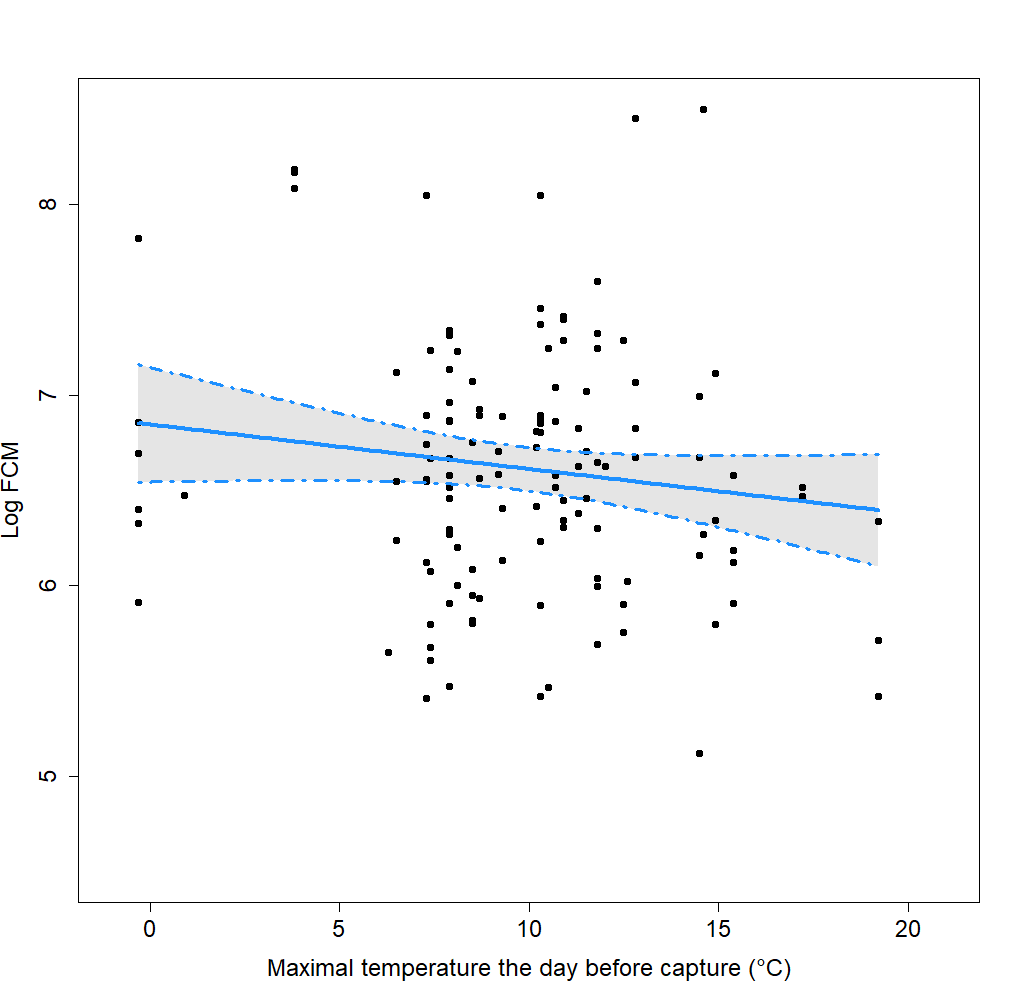


**Fig. 2** Relationship between FCMs level and maximal temperature the day before capture in the roe deer population of Aurignac. Points represent observed values, lines represent model predictions and dashed lines represent the 95% confidence interval.
